# Supplementary material for: The context-dependent epigenetic and organogenesis programs determine 3D vs. 2D cellular fitness of MYC-driven murine liver cancer cells
Source: eLife. 2025 May 6;14:RP101299. doi: 10.7554/eLife.101299 (PMC12055005; doi:10.7554/eLife.101299)
Supplement: Figure 3—source data 1. [file elife-101299-fig3-data1.zip › Figure 3L-source data 1/Fig3L.pdf]

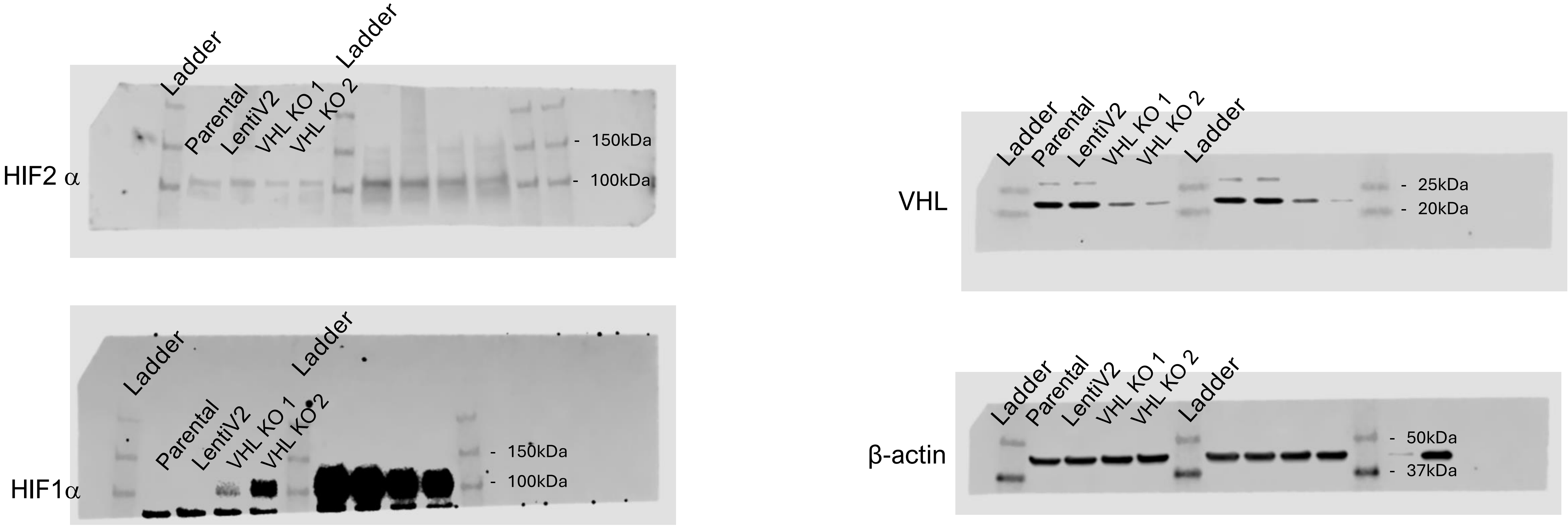

Figure 3L Source data. Original membranes corresponding to Figure 3, panel L. Western blot analysis of VHL CRISPR knockout in HepG2 cells using two gRNAs with HIF1a,Hi2a, VHL and β-actin.
