# Supplementary figures and images for: The context-dependent epigenetic and organogenesis programs determine 3D vs. 2D cellular fitness of MYC-driven murine liver cancer cells
Source: eLife. 2025 May 6;14:RP101299. doi: 10.7554/eLife.101299 (PMC12055005; doi:10.7554/eLife.101299)

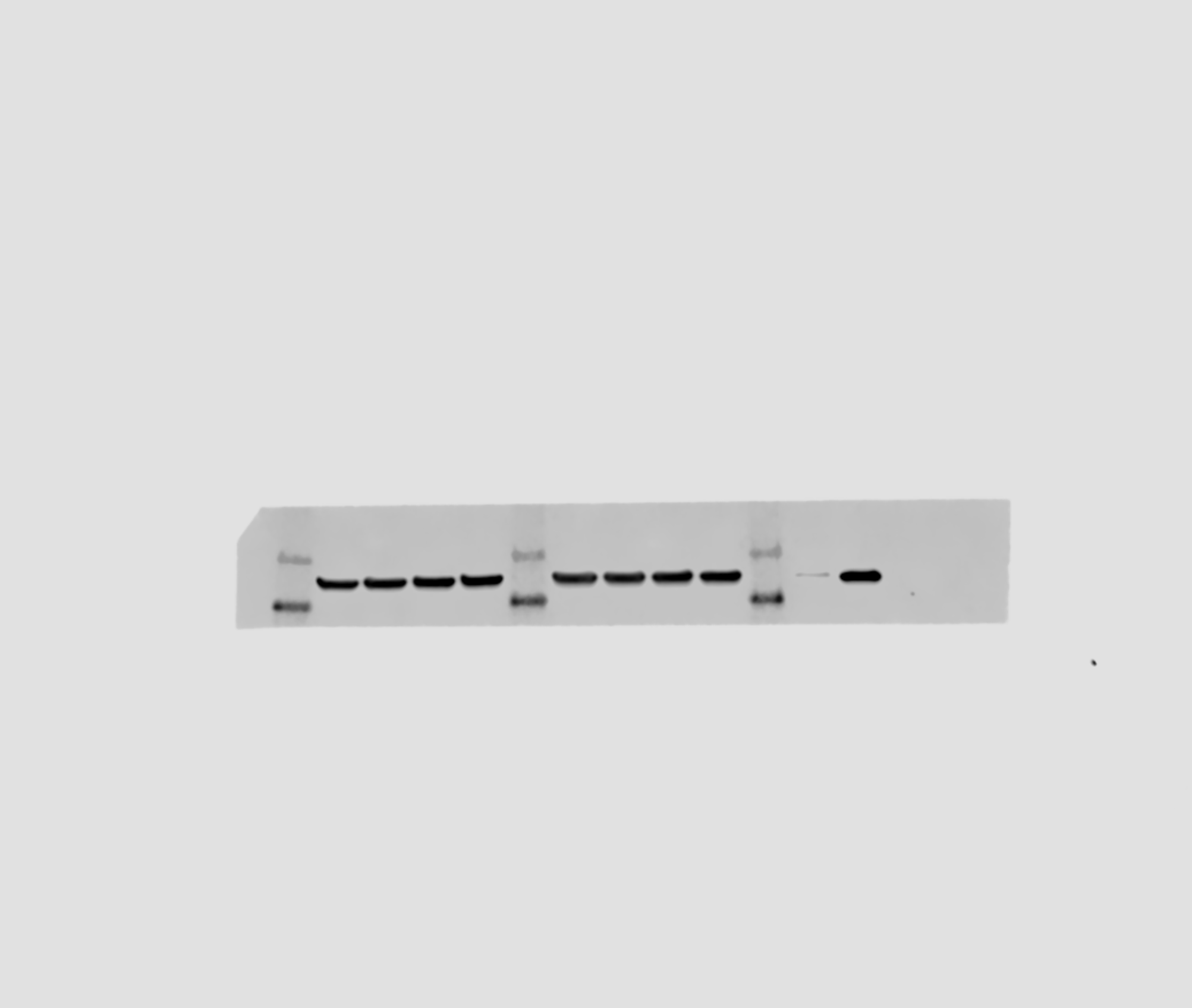

Supplement: Figure 3—source data 2. [file elife-101299-fig3-data2.zip › Figure 3L-source data 2/B-actin.tif]

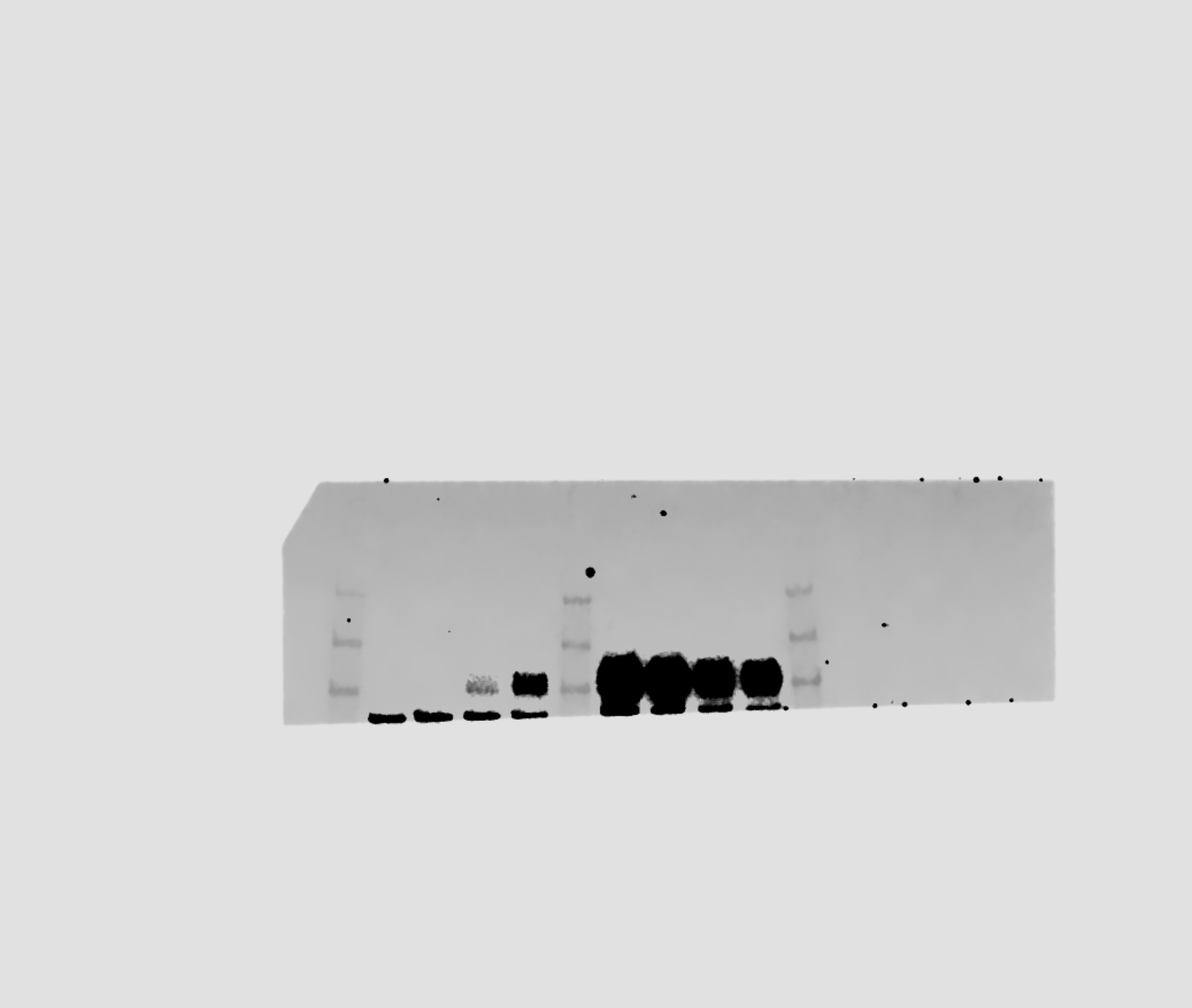

Supplement: Figure 3—source data 2. [file elife-101299-fig3-data2.zip › Figure 3L-source data 2/HIF1a.tif]

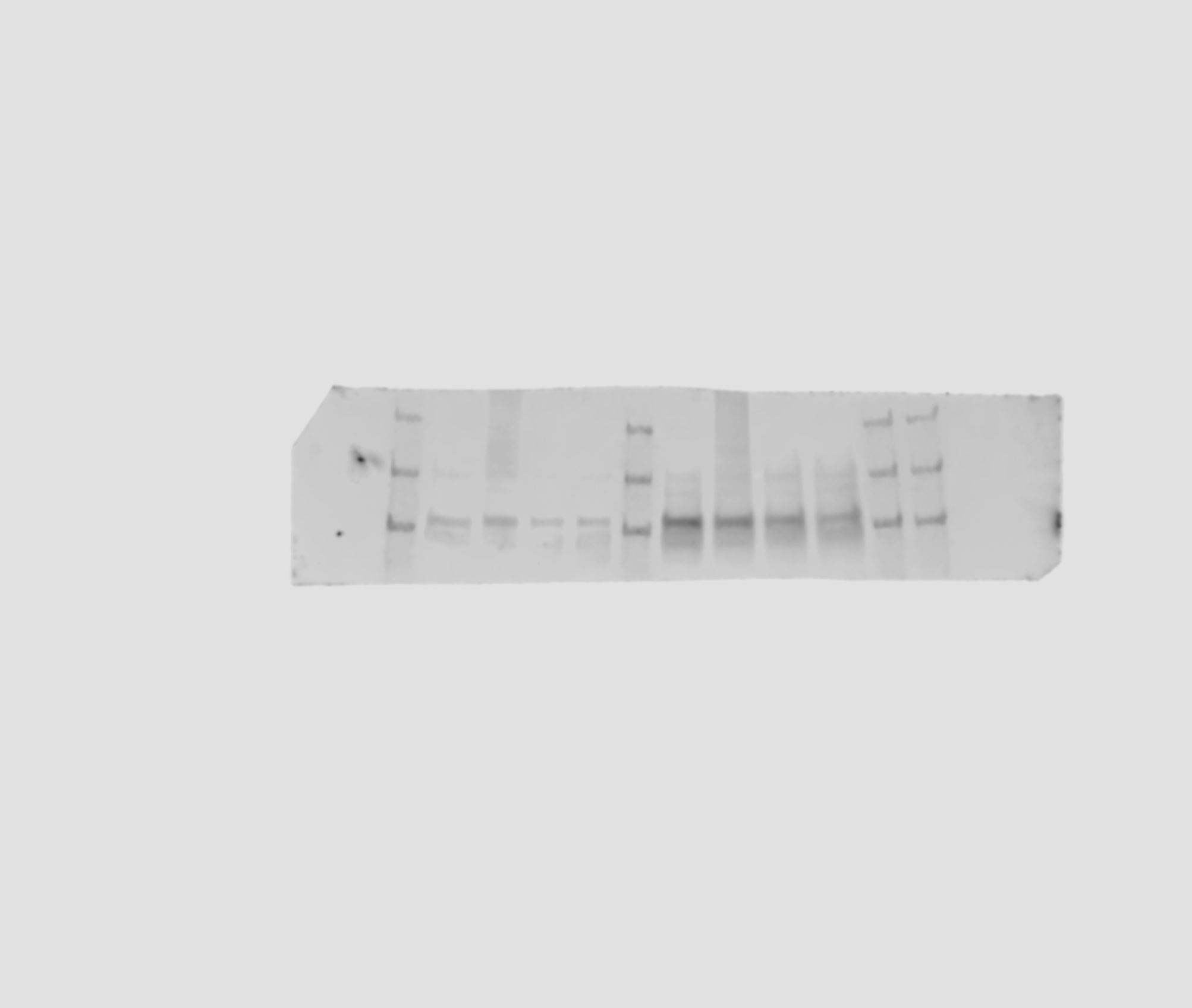

Supplement: Figure 3—source data 2. [file elife-101299-fig3-data2.zip › Figure 3L-source data 2/HIF2a.tif]

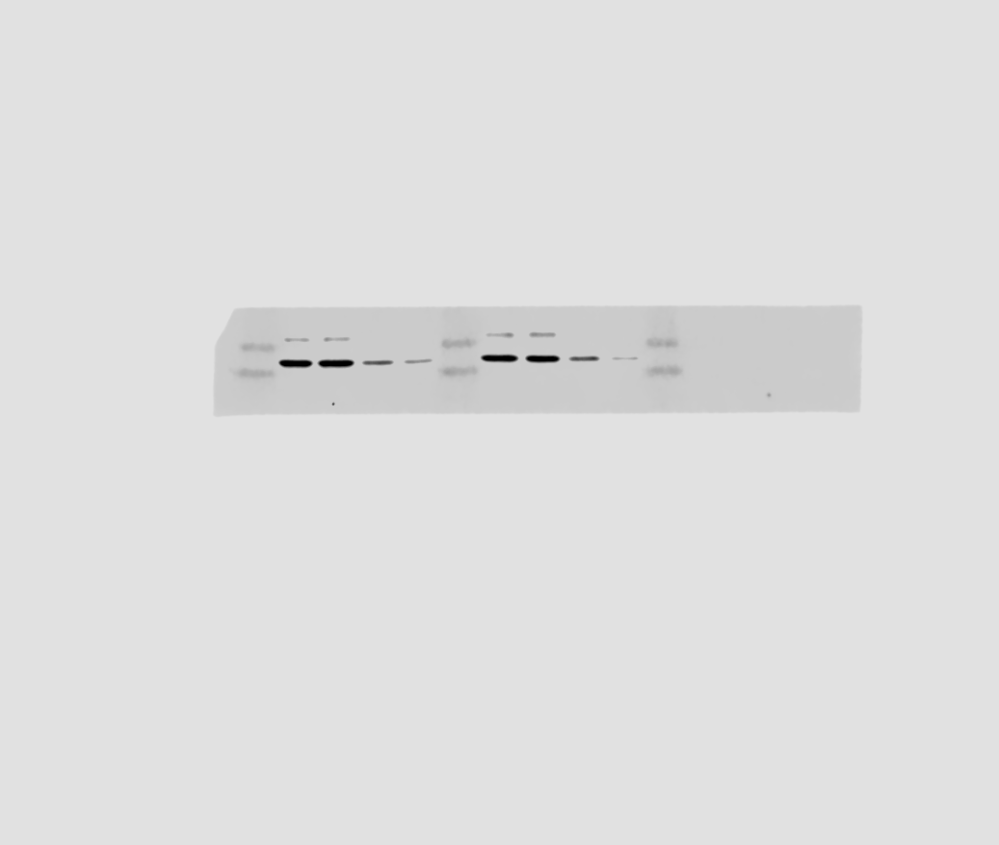

Supplement: Figure 3—source data 2. [file elife-101299-fig3-data2.zip › Figure 3L-source data 2/VHL.png]

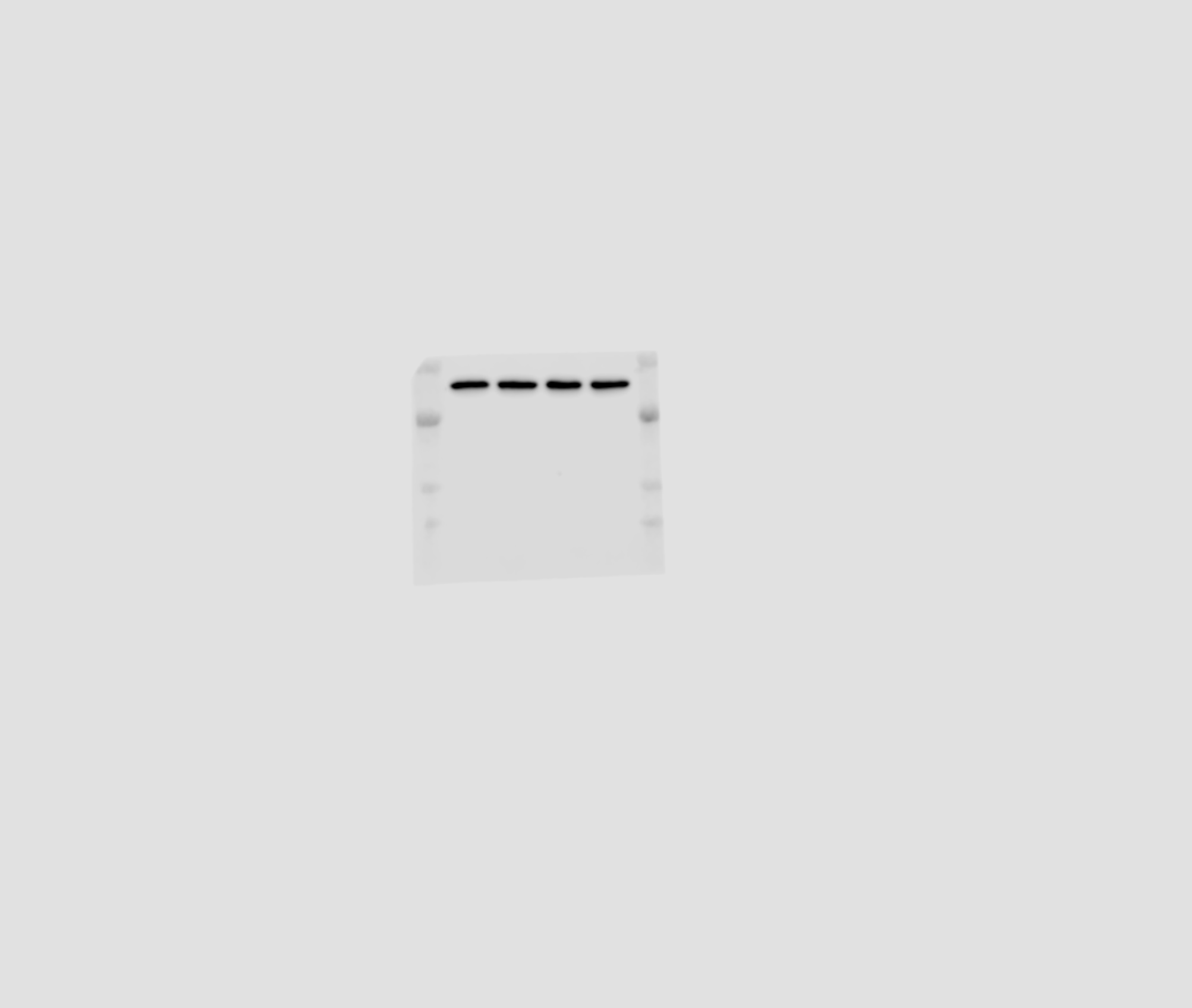

Supplement: Figure 7—source data 2. [file elife-101299-fig7-data2.zip › Figure 7B-source data 2/actin-NEJF10-shPRMT5.tif]

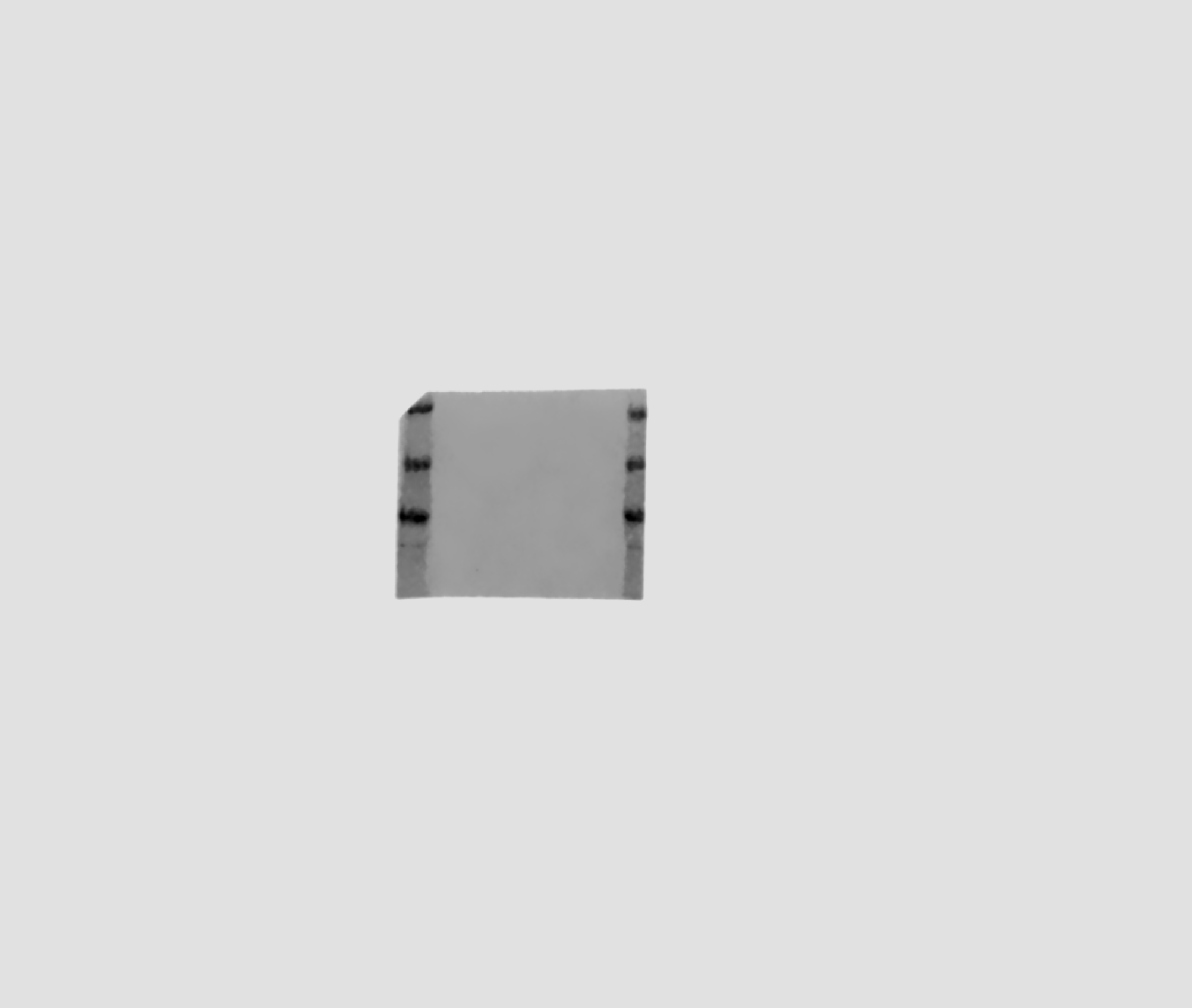

Supplement: Figure 7—source data 2. [file elife-101299-fig7-data2.zip › Figure 7B-source data 2/PRMT5-NEJF10-shPRMT5--Ladder only.tif]

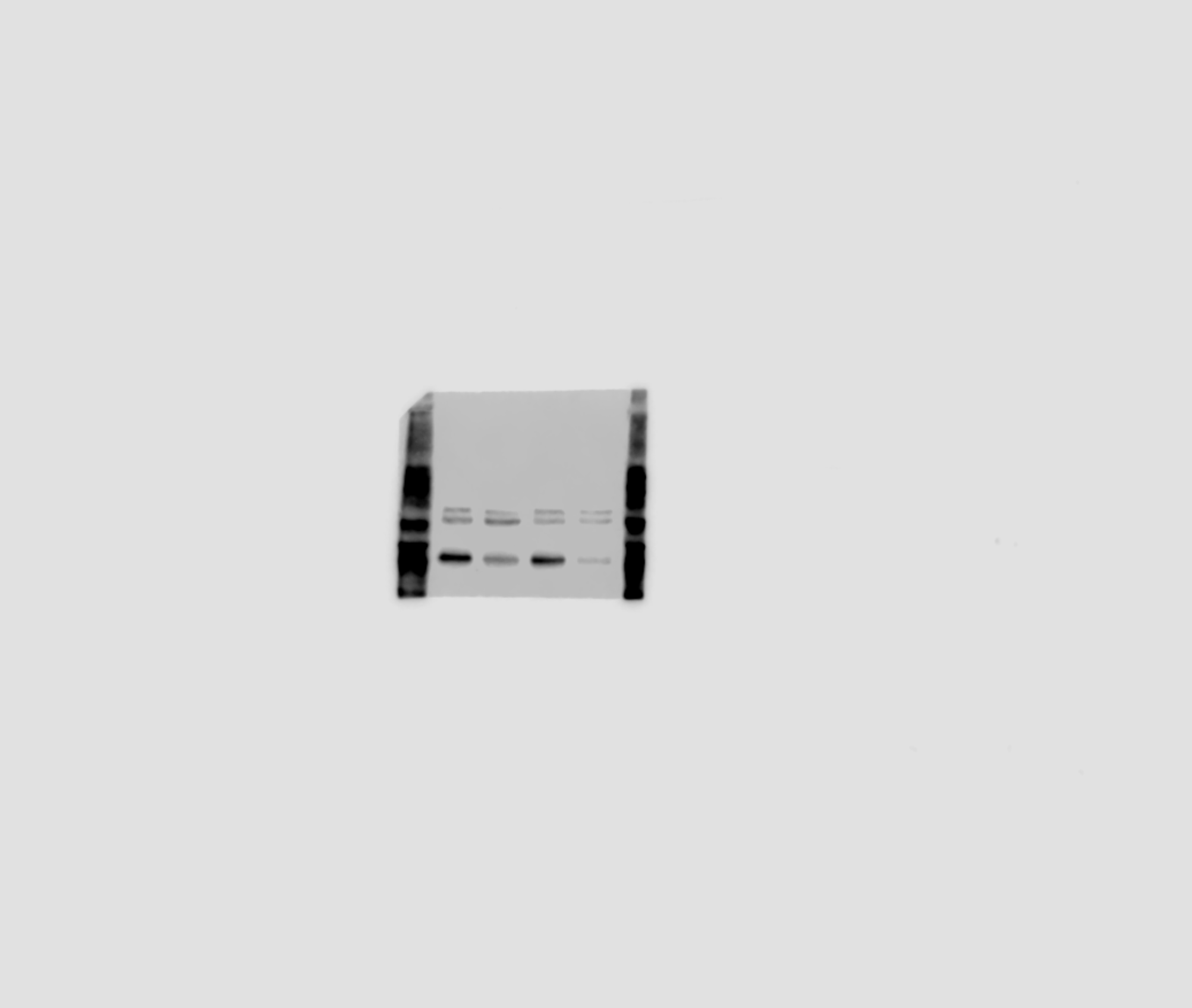

Supplement: Figure 7—source data 2. [file elife-101299-fig7-data2.zip › Figure 7B-source data 2/PRMT5-NEJF10-shPRMT5.tif]

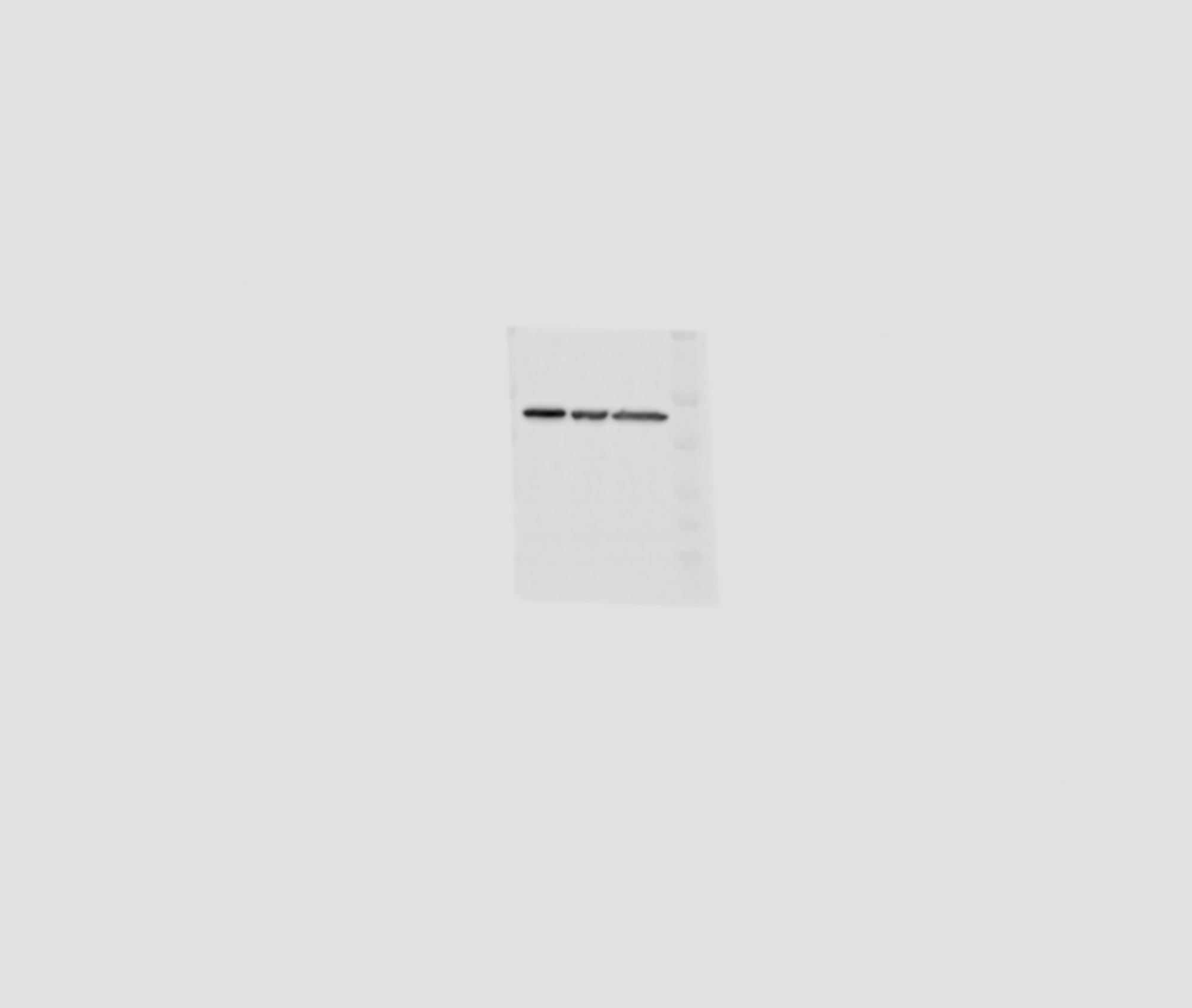

Supplement: Figure 7—source data 4. [file elife-101299-fig7-data4.zip › Figure 7J-source data 2/actin-NEJF10-2D-3D-7.tif]

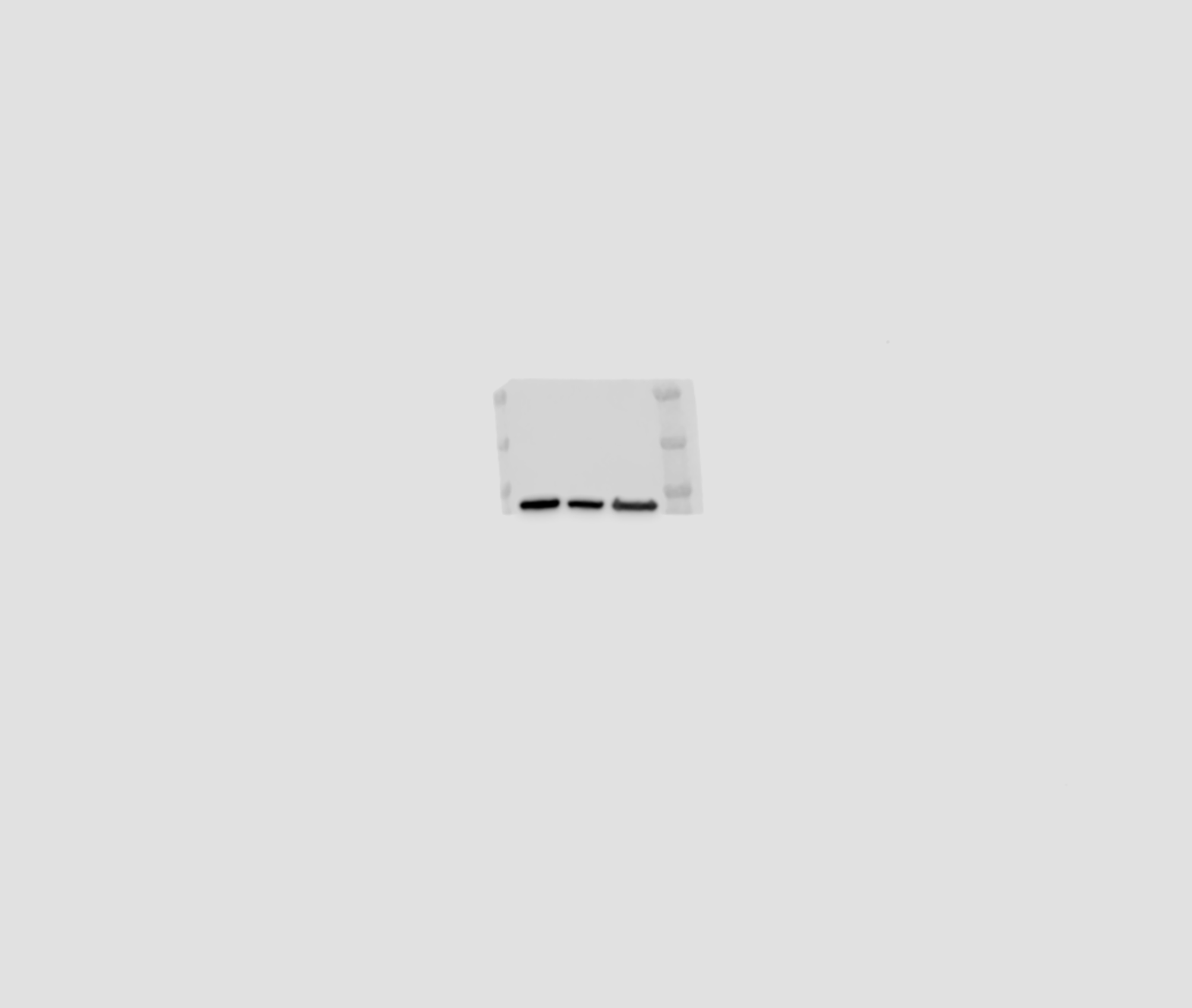

Supplement: Figure 7—source data 4. [file elife-101299-fig7-data4.zip › Figure 7J-source data 2/HSP90-NEJF10-2D-3D-7.tif]

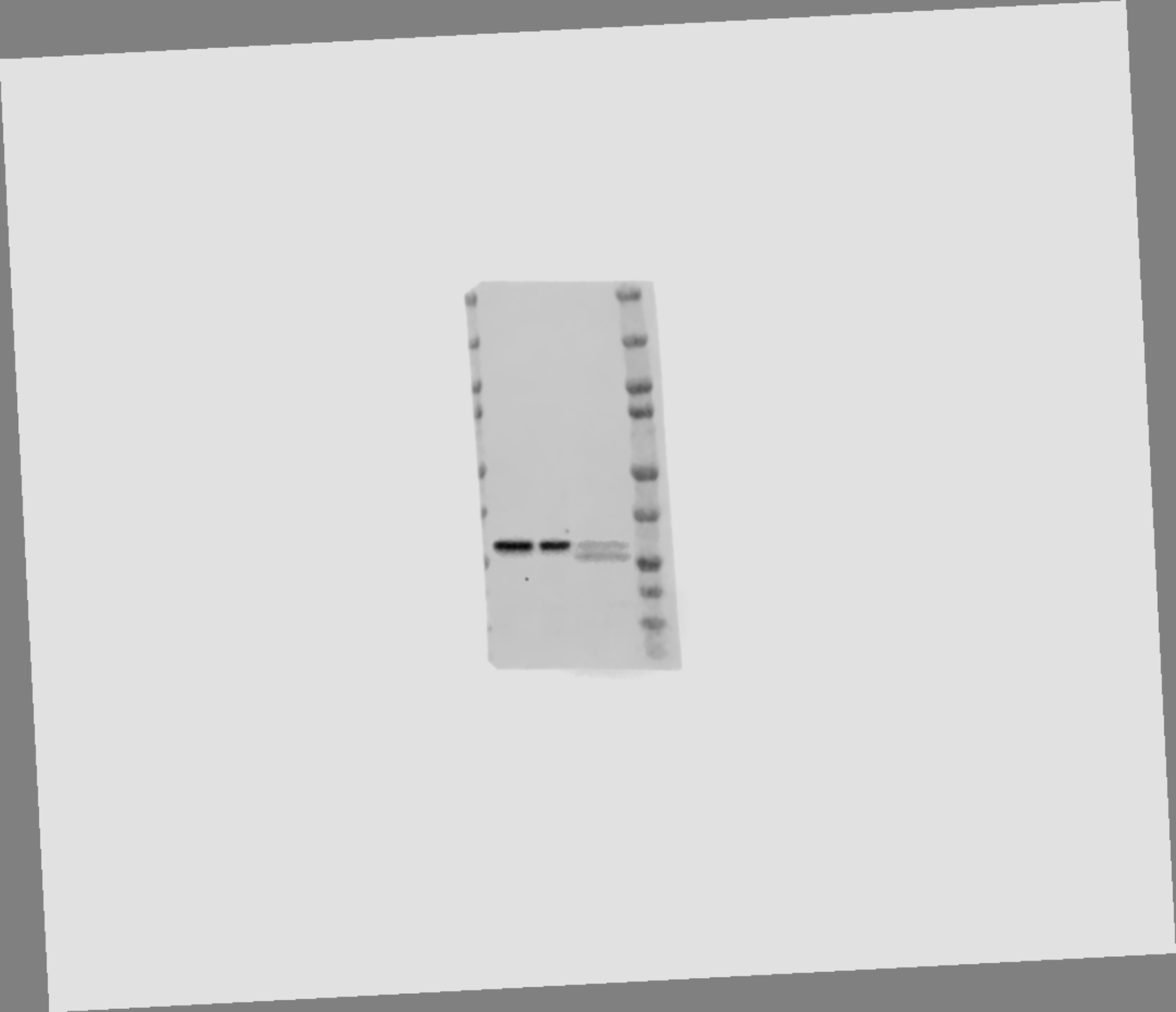

Supplement: Figure 7—source data 4. [file elife-101299-fig7-data4.zip › Figure 7J-source data 2/MTAP-NEJF10-2D-3D-7.tif.tif]
