# Supplementary material for: The context-dependent epigenetic and organogenesis programs determine 3D vs. 2D cellular fitness of MYC-driven murine liver cancer cells
Source: eLife. 2025 May 6;14:RP101299. doi: 10.7554/eLife.101299 (PMC12055005; doi:10.7554/eLife.101299)
Supplement: Figure 7—source data 1. [file elife-101299-fig7-data1.zip › Figure 7B-source data 1/Figure 7 B.pdf]

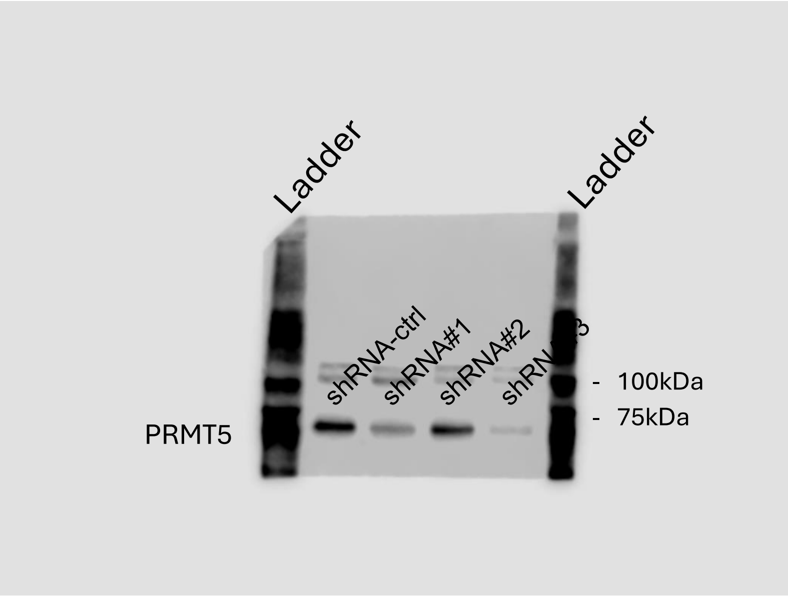

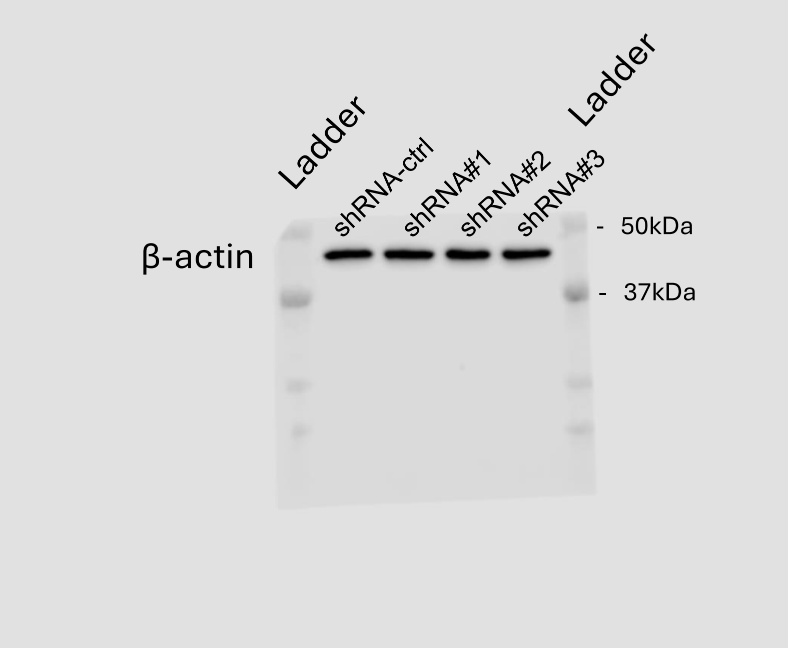

Figure 7B Source data. Original membranes corresponding to Figure 7, panel B. Western blot analysis with PRMT5 and β-actin antibodies after Prtm5 knockdown in NEJF10 cells.
