# Supplementary material for: The context-dependent epigenetic and organogenesis programs determine 3D vs. 2D cellular fitness of MYC-driven murine liver cancer cells
Source: eLife. 2025 May 6;14:RP101299. doi: 10.7554/eLife.101299 (PMC12055005; doi:10.7554/eLife.101299)
Supplement: Figure 7—source data 3. [file elife-101299-fig7-data3.zip › Figure 7J-source data 1/Figure 7 J.pdf]

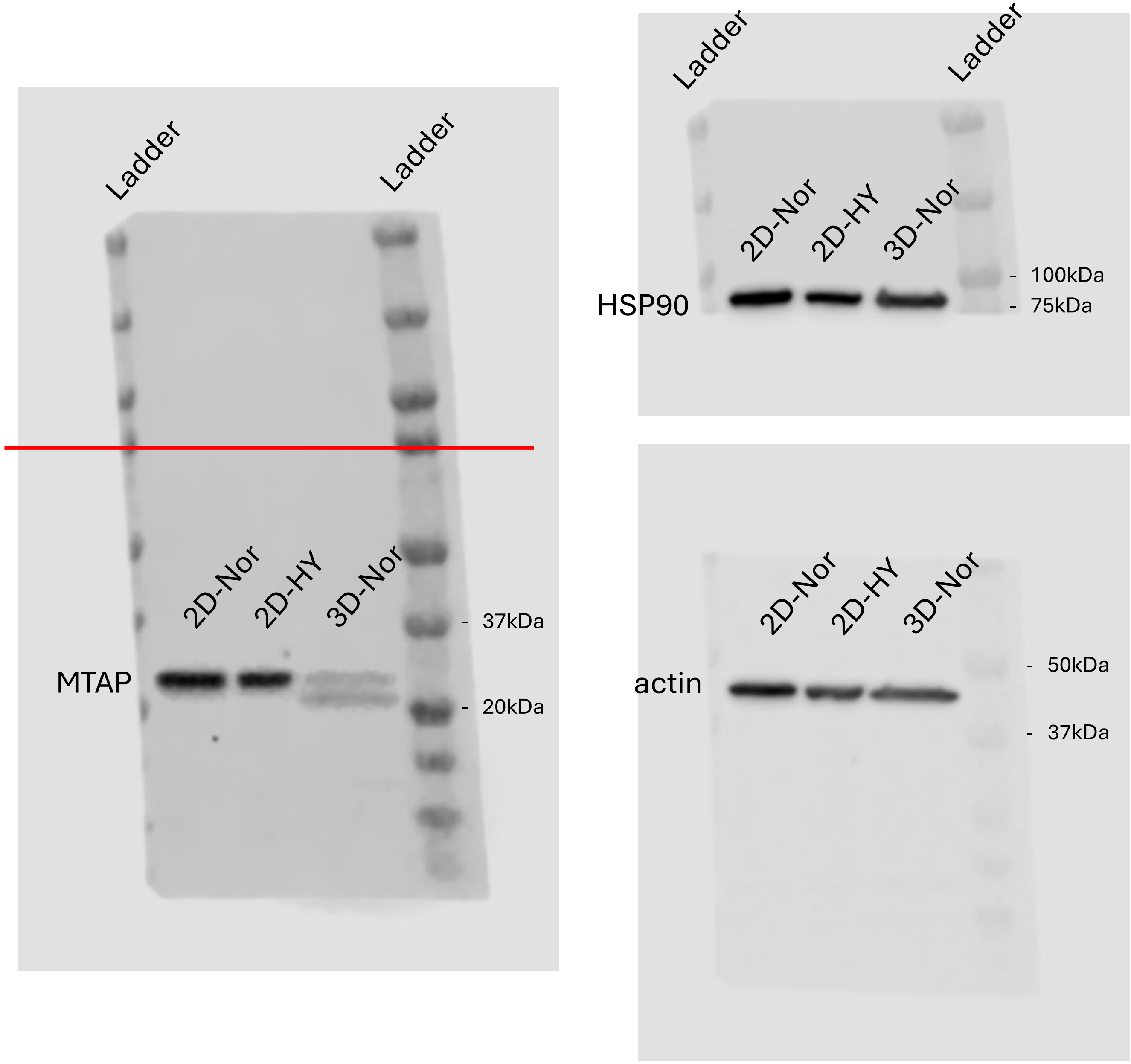

Figure 7J Source data. Original membranes corresponding to Figure 7, panel J. Western blot analysis MTAP, HSP90, and actin of NEJF10 whole cell lysates cultured from 2D normoxia (2D-Nor) and hypoxia (2D-HY) and 3D normoxia (3D-Nor) for 3 days.
Membrane was probed MTAP first. Then, membrane was cut cross red line into 2 membranes for probing HSP90 and actin.
